# Should all hospitalised patients colonised with *Candida auris* be considered for isolation?

**DOI:** 10.2807/1560-7917.ES.2024.29.45.2400729

**Published:** 2024-11-07

**Authors:** Eelco FJ Meijer, Andreas Voss

**Affiliations:** 1 Radboudumc-CWZ Center of Expertise for Mycology, Nijmegen, the Netherlands; 2 Department of Medical Microbiology and Immunology, Canisius-Wilhelmina Hospital/Dicoon, Nijmegen, the Netherlands; 3 Department of Medical Microbiology and Infection Control, University Medical Center Groningen, Groningen, the Netherlands

**Keywords:** Candida Candidozyma auris infection prevention guideline

*Candida auris* is an emerging pathogenic yeast causing nosocomial infections coupled with high mortality mainly in fragile people. It is considered a serious global health threat and classified as critical fungal pathogen on the World Health Organization (WHO) fungal priority pathogens list and listed on the WHO Global research agenda for antimicrobial resistance [1,2]. The organism's implications for patients and healthcare systems are high because of its potential for rapid resistance development and transmission within and between healthcare settings. The latter is furthered by the fact that *C. auris* is difficult to detect under routine laboratory conditions. In addition, decolonisation of patients is challenging and mortality rates in those infected are high [3,4].

In this issue of *Eurosurveillance*, Meletiadis et al. [5] describe the prolonged occurrence of *Candida auris* in a tertiary care hospital in Greece, leading to recurrent waves with increased numbers of colonised patients, followed by increased morbidity due to bloodstream infections. Not only did authors confirm clonality of the involved isolates, but they also demonstrated the emergence of echinocandin resistance over time. Noting how the lack of strict and continuous implementation of infection prevention and control (IPC) measures affected the course of the outbreak, Meletiadis et al. stress the need to implement stringent IPC measures immediately during first detection/introduction of *C. auris* and underscore the importance of adequate diagnostics and surveillance. This is of utmost importance for preventing and controlling the spread of *C. auris* and other multidrug-resistant organisms (MDRO) within and between hospitals.

In the Netherlands, a revised national MDRO guideline was published on 11 October 2024 [6]. This version by the Dutch Collaborative Partnership for Infection Prevention Guidelines (SRI, https://www.sri-richtlijnen.nl) includes *C. auris* for which it was decided that it is justified to isolate all hospitalised patients colonised with *C. auris* using stringent IPC measures. This decision followed an elaborate discussion, whether or not it is justified to handle patients with resistant and susceptible isolates of *C. auris* in the same way.

The Dutch hospital setting is renowned for its robust and successful IPC approach. Combined with the national antimicrobial stewardship programme, it has kept the country’s antimicrobial resistance rates at one of the lowest levels in Europe [7]. One outstanding example of this IPC approach is the early adaptation in 1986 of the ‘search and destroy’ strategy to control meticillin-resistant *Staphylococcus aureus* (MRSA) [8]. A comparable ‘search and contain’ strategy was nationally implemented in 2012, targeting other MDROs, including carbapenem-resistant Enterobacterales, which were emerging in the Netherlands at the time [9]. Currently, the prevalence for MRSA and carbapenem-resistant *Klebsiella pneumonia* are among the lowest in Europe, at 1.8% and 0.4%, respectively [7].

The first Dutch guideline for MDRO was published in 2005, with the latest revision initiated by the SRI in 2022 [6]. During the concept and revision phase of this guideline, far-reaching IPC measures were initially advised for patients harbouring resistant and susceptible *C. auris* isolates [10], leading to the above-mentioned discussion among experts whether susceptible isolates of *C. auris* warrant isolation measures to the same extent as resistant ones. Opponents argued that by definition, a susceptible isolate is not multidrug resistant and therefore should not be treated equally to the resistant variant. Consequently, they suggested less stringent measures for patients colonised or infected with susceptible isolates of *C. auris*, an approach in line with current practice for resistant Enterobacterales and their susceptible counterparts.

However, following the considerations elaborated further on, members of the MDRO working group reached the final consensus to handle all *C. auris* isolates equally. Consequently, all patients colonised or infected with *C. auris*, independent of their susceptibility testing outcome, will be cared for in a single-bed isolation room with an anteroom and adequate ventilation for source isolation. Although an isolation room may seem an excessive measure given the contact-based transmission route of *C. auris*, it was chosen to enhance compliance with the IPC measures by creating extra attention. The guideline advises gown, gloves and masks for all healthcare workers entering the room and stress the need for thorough room cleaning and adequate hand disinfection.

Considering the experiences and observations by Meletiadis et al., the decision to align the prevention of *C. auris* transmission regardless of its antifungal resistance will aid to avert *C. auris* outbreaks and the pathogen from becoming endemic in Dutch hospitals. The following reasoning for the Dutch decision may inform decision-making by others who are facing similar considerations.

*Candida auris* has demonstrated a notable propensity for rapid resistance development and is the first *Candida* species to have acquired pan-drug resistance [11]. Even during short term echinocandin treatment, the first-line treatment for systemic *C. auris* infections, resistance development has been demonstrated during the outbreak described by Meletiadis et al. [5] and unequivocally proven in a patient using whole genome sequencing [12]. For azoles and other antifungal drugs, resistance can arise via multiple mechanisms, including acquired (point) mutations and the upregulation of resistance genes [13,14]. Consequently, patients colonised with initially susceptible strains could harbour or develop resistance during treatment, underscoring the necessity of rigorous infection control measures.

Well-known for its capacity to spread within healthcare settings, *C. auris* has often led to outbreaks that are challenging to contain [3,5]. Standard cleaning protocols typically used for bacterial MDROs have proven inadequate for eradicating *C. auris* from the healthcare environment [15]. Its combined properties, such as biofilm formation and salt- and thermotolerance, facilitate prolonged patient colonisation and survival outside the host resembling the outbreak potential of *C. parapsilosis*, another globally impactful yeast [16,17]. Patients colonised with *C. auris* serve as reservoirs for transmission to other patients and healthcare workers [18]. Notably, pan-susceptible isolates have led to a multicenter outbreak in Brazil, demonstrating how its pathogenicity and outbreak potential is not solely driven by its antifungal resistance [19]. Isolating all patients, irrespective of the antifungal susceptibility pattern of the colonising isolate, will significantly reduce the risk of transmission within healthcare facilities, thereby averting outbreaks.

*Candida auris* is challenging to detect accurately using conventional diagnostic methods on samples from routinely screened patient body sites, which may result in underestimation of colonisation rates and delayed implementation of IPC measures [18,20]. Therefore, additional axilla and groin swabs along with qPCR testing are recommended, although PCR-positive samples have proven difficult to culture [20,21]. Fortunately, the recent identification of novel genetic variants (clades) has not impeded molecular detection thus far [11,22,23]. However, decolonisation strategies for patients with *C. auris* have been largely ineffective, further emphasising the importance of preventive measures, such as isolation, to restrict the healthcare-associated spread of this yeast, particularly in non-endemic areas [4,24].

Invasive infections caused by *C. auris* and other emerging *Candida* species, are associated with even higher mortality rates (exceeding 46 %) than the most commonly encountered *Candida* species in invasive disease, particularly among vulnerable patient populations [25,26]. A survey from 2022 on reported cases of *Candida auris* infection or carriage, including hospital outbreaks, has demonstrated invasive infections in over 10 % of colonised patients in the European setting [27], and colonisation has occurred within as little as 4 hours of contact [28]. Therefore, preventing the spread of colonisation, even if the strain is initially susceptible to treatment, is crucial to safeguard patient health and minimise the risk of invasive infections.

Adopting a proactive approach by isolating all colonised patients aligns with the principles of infection prevention and control. By implementing comprehensive isolation measures across healthcare settings, facilities can effectively contain the spread of *C. auris* and mitigate associated risks, including treatment resistance and healthcare-associated infections. With regards to financial impact, preventing a prolonged outbreak lasting over 10 months, and involving 36 patients who became colonised at the hospital including eight with BSI in a tertiary care center in London in 2016, would have saved an estimated 1 million GBP (1.2 million EUR) [29].

Even within yeasts, *C. auris* is in a league of its own. Prioritising patient safety through comprehensive infection prevention and control measures enables healthcare facilities to manage the risks associated with *C. auris* colonisation, thereby minimising the likelihood of outbreaks and invasive infections. While *C. auris* imported cases and outbreaks are increasing in Europe [27], no *C. auris* outbreaks have been reported in the Netherlands to date, although importations in the Netherlands are gradually increasing [20]. Aside from encouraging stringent IPC in Dutch policymaking, the Dutch National Reference Center for Mycology has requested that all laboratories submit isolates for antifungal susceptibility testing and genotyping to monitor transmission. Continued surveillance for fungal pathogens remains key. Other countries may consider similar approaches and endorse experts to share lessons learned in containing the spread of *C. auris*.

## Note

Eelco Meijer is the head of the Dutch National Reference Center for Mycology, location Canisius-Wilhelmina Hospital, Nijmegen, the Netherlands. He partakes in (inter)national discussions and guidelines on *Candida auris* and is project leader of the ZonMw Consortium CAUTION: *Candida auris* screening, surveillance and infection control project No:10150022310025.

Andreas Voss is Chair of the Dutch Collaborative Partnership for Infection Prevention Guidelines (SRI), which published the Dutch MDRO guideline in October 2024.

## References

[1] World Health Organization (WHO). WHO fungal priority pathogens list to guide research, development and public health action. Geneva: WHO. 25 Oct 2022. Available from: https://www.who.int/publications/i/item/9789240060241

[2] World Health Organization (WHO). Global research agenda for antimicrobial resistance in human health. Geneva: WHO. 22 Jun 2023. Available from: https://www.who.int/publications/m/item/global-research-agenda-for-antimicrobial-resistance-in-human-health

[3] Kenters N Kiernan M Chowdhary A Denning DW Pemán J Saris K Control of Candida auris in healthcare institutions: Outcome of an International Society for Antimicrobial Chemotherapy expert meeting. Int J Antimicrob Agents. 2019;54(4):400-6. 10.1016/j.ijantimicag.2019.08.013 31419480

[4] Rosa R Abbo LM Jimenez A Carter C Ruiz M Gerald W Effectiveness of a sodium hypochlorite isotonic solution in decolonization of patients with Candida auris: Learnings from a county health care system. Am J Infect Control. 2024;52(5):595-8. 10.1016/j.ajic.2023.11.008 38007101

[5] Meletiadis J Siopi M Spruijtenburg B Georgiou PC Kostoula M Vourli S Candida auris fungaemia outbreak in a tertiary care academic hospital and emergence of a pan-echinocandin resistant isolate, Greece, 2021 to 2023. Euro Surveill. 2024;29(45):2400128. 10.2807/1560-7917.ES.2024.29.45.2400128 PMC11258949 39027938

[6] Samenwerkingsverband Richtlijnen Infectiepreventie (SRI). Bijzonder resistente micro-organismen (BRMO). [Dutch Collaborative Partnership for Infection Prevention Guidelines (SRI). Multidrug-resistant organisms (MDRO)]. Bilthoven: SRI; 11 Oct 2024. Dutch. Available from: https://www.sri-richtlijnen.nl/brmo

[7] European Centre for Disease Prevention and Control (ECDC). Surveillance Atlas of Infectious Diseases. Stockholm: ECDC. 28 Apr 2023. Available from: https://www.ecdc.europa.eu/en/surveillance-atlas-infectious-diseases

[8] Wertheim HF Vos MC Boelens HA Voss A Vandenbroucke-Grauls CM Meester MH Low prevalence of methicillin-resistant Staphylococcus aureus (MRSA) at hospital admission in the Netherlands: the value of search and destroy and restrictive antibiotic use. J Hosp Infect. 2004;56(4):321-5. 10.1016/j.jhin.2004.01.026 15066745

[9] Overdevest I, Nijs M, Op den Buijs I, van Dommelen L, Fonville J. Optimalisatie van screening op carbapenemase-producerende Enterobacteriaceae. [Screening optimalisation for Carbapenemase-producing Enterobacterales]. Ned Tijdschr Med Microbiol. 2019;272Dutch. Available from: https://www.nvmm.nl/ntmm/artikeloverzicht/juni-2019

[10] Samenwerkingsverband Richtlijnen Infectiepreventie (SRI). Conceptrichtlijn 10 Infectiepreventie Bijzonder Resistente MicroOrganismen (BRMO). [Dutch Collaborative Partnership for Infection Prevention Guidelines (SRI). Concept guideline 10 infection prevention multidrug-resistant organisms (MDRO)]. Bilthoven: SRI. [Accessed: 25 Jul 2024]. Dutch. Available from: https://www.nvmm.nl/media/5032/conceptrichtlijn-infectiepreventie-bijzonder-resistente-micro-organismen-brmo-_def-versie-3.pdf

[11] Lockhart SR Etienne KA Vallabhaneni S Farooqi J Chowdhary A Govender NP Simultaneous Emergence of Multidrug-Resistant Candida auris on 3 Continents Confirmed by Whole-Genome Sequencing and Epidemiological Analyses. Clin Infect Dis. 2017;64(2):134-40. 10.1093/cid/ciw691 27988485 PMC5215215

[12] Spruijtenburg B Ahmad S Asadzadeh M Alfouzan W Al-Obaid I Mokaddas E Whole genome sequencing analysis demonstrates therapy-induced echinocandin resistance in Candida auris isolates. Mycoses. 2023;66(12):1079-86. 10.1111/myc.13655 37712885

[13] Carolus H Pierson S Muñoz JF Subotić A Cruz RB Cuomo CA Genome-Wide Analysis of Experimentally Evolved Candida auris Reveals Multiple Novel Mechanisms of Multidrug Resistance. MBio. 2021;12(2):e03333-20. 10.1128/mBio.03333-20 33820824 PMC8092288

[14] Rybak JM Barker KS Muñoz JF Parker JE Ahmad S Mokaddas E In vivo emergence of high-level resistance during treatment reveals the first identified mechanism of amphotericin B resistance in Candida auris. Clin Microbiol Infect. 2022;28(6):838-43. 10.1016/j.cmi.2021.11.024 34915074 PMC9467277

[15] Cadnum JL Shaikh AA Piedrahita CT Sankar T Jencson AL Larkin EL Effectiveness of Disinfectants Against Candida auris and Other Candida Species. Infect Control Hosp Epidemiol. 2017;38(10):1240-3. 10.1017/ice.2017.162 28793937

[16] Welsh RM Bentz ML Shams A Houston H Lyons A Rose LJ Survival, Persistence, and Isolation of the Emerging Multidrug-Resistant Pathogenic Yeast Candida auris on a Plastic Health Care Surface. J Clin Microbiol. 2017;55(10):2996-3005. 10.1128/JCM.00921-17 28747370 PMC5625385

[17] Daneshnia F de Almeida Júnior JN Ilkit M Lombardi L Perry AM Gao M Worldwide emergence of fluconazole-resistant Candida parapsilosis: current framework and future research roadmap. Lancet Microbe. 2023;4(6):e470-80. 10.1016/S2666-5247(23)00067-8 37121240 PMC10634418

[18] Sansom SE Gussin GM Schoeny M Singh RD Adil H Bell P Rapid Environmental Contamination With Candida auris and Multidrug-Resistant Bacterial Pathogens Near Colonized Patients. Clin Infect Dis. 2024;78(5):1276-84. 10.1093/cid/ciad752 38059527 PMC11093678

[19] Spruijtenburg B Nobrega de Almeida Júnior J Ribeiro FC Kemmerich KK Baeta K Meijer EFJ Multicenter Candida auris outbreak caused by azole-susceptible clade IV in Pernambuco, Brazil. Mycoses. 2024;67(6):e13752. 10.1111/myc.13752 38880933

[20] Leonhard SE Chong GM Foudraine DE Bode LGM Croughs P Popping S Proposal for a screening protocol for Candida auris colonization. J Hosp Infect. 2024;146:31-6. 10.1016/j.jhin.2023.12.019 38286238

[21] Centers for Disease Control and Prevention (CDC). C. auris Screening: Patient Swab Collection. Atlanta: CDC; 24 Apr 2024. Available from: https://www.cdc.gov/candida-auris/hcp/screening-hcp/c-auris-screening-patient-swab-collection-1.html

[22] Spruijtenburg B Badali H Abastabar M Mirhendi H Khodavaisy S Sharifisooraki J Confirmation of fifth Candida auris clade by whole genome sequencing. Emerg Microbes Infect. 2022;11(1):2405-11. 10.1080/22221751.2022.2125349 36154919 PMC9586689

[23] Khan T Faysal NI Hossain MM Mah-E-Muneer S Haider A Moon SB Emergence of the novel sixth Candida auris Clade VI in Bangladesh. Microbiol Spectr. 2024;12(7):e0354023. 10.1128/spectrum.03540-23 38842332 PMC11218448

[24] Meijer EFJ, Voss A, Meis JF. Candida auris: huidige inzichten. [Candida auris: current insights]. Ned Tijdschr Med Microbiol. 2022;302Dutch. Available from: https://www.nvmm.nl/media/4649/19962-061.pdf

[25] Alfouzan W Ahmad S Dhar R Asadzadeh M Almerdasi N Abdo NM Molecular Epidemiology of Candida Auris Outbreak in a Major Secondary-Care Hospital in Kuwait. J Fungi (Basel). 2020;6(4):307. 10.3390/jof6040307 33233388 PMC7712429

[26] Hoenigl M Salmanton-García J Egger M Gangneux JP Bicanic T Arikan-Akdagli S Guideline adherence and survival of patients with candidaemia in Europe: results from the ECMM Candida III multinational European observational cohort study. Lancet Infect Dis. 2023;23(6):751-61. 10.1016/S1473-3099(22)00872-6 37254300

[27] Kohlenberg A Monnet DL Plachouras D Candida auris survey collaborative group Candida auris survey collaborative group includes the following national experts . Increasing number of cases and outbreaks caused by Candida auris in the EU/EEA, 2020 to 2021. Euro Surveill. 2022;27(46):2200846. 10.2807/1560-7917.ES.2022.27.46.2200846 36398575 PMC9673237

[28] Schelenz S Hagen F Rhodes JL Abdolrasouli A Chowdhary A Hall A First hospital outbreak of the globally emerging Candida auris in a European hospital. Antimicrob Resist Infect Control. 2016;5(1):35. 10.1186/s13756-016-0132-5 27777756 PMC5069812

[29] Taori SK Khonyongwa K Hayden I Athukorala GDA Letters A Fife A Candida auris outbreak: Mortality, interventions and cost of sustaining control. J Infect. 2019;79(6):601-11. 10.1016/j.jinf.2019.09.007 31557493

